# Effect of GOAL-Directed ANalgesia using ANI (Analgesia/Nociception Index) during general anesthesia on immediate postoperative pain and intraoperative hemodynamics in adult patients (GOALDAN study): a study protocol for randomized, controlled, multicenter trial

**DOI:** 10.1186/s13063-022-06273-1

**Published:** 2022-04-25

**Authors:** Adrien Michalot, Jean-Étienne Bazin, Philippe Richebé, Bernard Allaouchiche, Emmanuel Boselli

**Affiliations:** 1 Department of Anesthesiology and Intensive Care, University Hospital of Clermont-Ferrand, Clermont-Ferrand, France; 2 Department of Anesthesiology and Pain Medicine, Maisonneuve-Rosemont Hospital - CIUSSS de L’Est de l’Ile de Montréal, Université de Montréal, Montréal, Québec Canada; 3 APCSe VetAgro Sup UPSP 2016.A101, Claude Bernard Lyon 1 University, Université de Lyon, Marcy-l’Étoile, France; 4 Groupement Hospitalier Nord Dauphiné, Pierre Oudot Hospital Centre, Department of Anesthesiology, Bourgoin-Jallieu, France

**Keywords:** Anesthesia and analgesia, Intraoperative neurophysiological monitoring, Remifentanil, Randomized controlled trial, Pain, Postoperative

## Abstract

**Background:**

Severe postoperative pain remains a major problem that is seen in 20 to 40% of patients. The Analgesia/Nociception Index (ANI) is a 0–100 index reflecting the relative parasympathetic activity allowing for intraoperative analgesia monitoring. We have previously shown that an ANI value < 50 immediately before extubation may predict the occurrence of immediate postoperative pain with good performance. We hypothesized that GOAL-Directed ANalgesia may provide reduced immediate postoperative pain and optimized intraoperative remifentanil administration (GOLDAN study).

**Methods:**

The GOALDAN study is an international, multicenter, simple-blind, parallel, prospective, randomized, controlled, two-armed trial. Patients are randomly assigned in a 1:1 ratio in the control group or in the experimental group. Patients will be randomly allocated to either the intervention group (ANI) or the control group (standard care only). In the ANI group, the administration of remifentanil will be goal-directed targeting a 50–80 ANI range, with a prophylactic injection of morphine immediately after extubation if the case of ANI < 50. Our primary objective was to determine whether the prophylactic administration of morphine at the end of the procedure in patients at risk of immediate postoperative pain (ANI < 50 immediately before extubation) could reduce the incidence of the latter by 50% in the post-anesthetic care unit. Our secondary objective was to determine whether the intraoperative use of goal-directed analgesia with an ANI target of 50 to 80 could improve intraoperative hemodynamics and postoperative outcome.

**Discussion:**

Because of the paucity of well-conducted trials, the authors believe that a randomized-controlled trial will improve the evidence for using analgesia monitoring during general anesthesia and strengthen current recommendations for intraoperative analgesia management.

**Trial registration:**

ClinicalTrials.gov NCT03618082. Registered on 7 August 2018

## Administrative information

Note: The numbers in curly brackets in this protocol refer to SPIRIT checklist item numbers. The order of the items has been modified to group similar items (see https://nam12.safelinks.protection.outlook.com/?url=http%3A%2F%2Fwww.equator-network.org%2Freporting-guidelines%2Fspirit-2727-statement-defining-standard-protocol-items-for-clinical-trials%2F&data=04%7C01%7C%7Ccebfabce35fe40e5116c08da0ee97648%7C84df9e7fe9f640afb435aaaaaaaaaaaa%7C1%7C0%7C637838693113378492%7CUnknown%7CTWFpbGZsb3d8eyJWIjoiMC4wLjAwMDAiLCJQIjoiV2luMzIiLCJBTiI6Ik1haWwiLCJXVCI6Mn0%3D%7C3000&sdata=aaHdGNuTEzSHq%2FuqF5QPWlcD%2Fk3o7RX5kAxe%2FUb0WIw%3D&reserved=0).

**Table:** 

Title {1}	Effect of GOAL-Directed ANalgesia using ANI (Analgesia/Nociception Index) during general anesthesia on immediate postoperative pain and intraoperative hemodynamics in adult patients (GOALDAN study): a study protocol for randomized, controlled, multicentre trial
Trial registration {2a and 2b}	Registered on 7 August 2018 at https://clinicaltrials.gov/ (trial number NCT 03618082)
Protocol version {3}	Version 4.0, June 2019
Funding {4}	This study was funded by institutional support and by a grant from the APICIL Foundation.
Author details {5a}	Adrien Michalot,^1^ Jean-Étienne Bazin,^1^ Philippe Richebé,^2^ Bernard Allaouchiche,^3,4^ Emmanuel Boselli^3,4^ 1. Department of Anesthesiology and Intensive Care, University Hospital of Clermont-Ferrand, Clermont-Ferrand, France 2. Department of Anesthesiology and Pain Medicine, Maisonneuve-Rosemont Hospital - CIUSSS de L’Est de l’Ile de Montréal, Université de Montréal, Montréal, Québec, Canada. 3. APCSe VetAgro Sup UPSP 2016.A101, Claude Bernard Lyon 1 University, Université de Lyon, Marcy-l’Étoile, France 4. Groupement Hospitalier Nord Dauphiné, Pierre Oudot Hospital Centre, Department of Anesthesiology, Bourgoin-Jallieu, France
Name and contact information for the trial sponsor {5b}	Dominique Morand, Clinical Research Associate (CRA), Clermont-Ferrand University Hospital, dmorand@chu-clermontferrand.fr
Role of sponsor {5c}	This is an investigator-initiated study. The study sponsor is the university hospital where the chief investigator is employed. The sponsor provides administrative, logistic, and other supports that are required for this study.

## Introduction

### Background and rationale {6a}

Severe postoperative pain remains a major problem that is seen in 20 to 40% of patients [1]. Numerous minor or average surgical procedures, some of which are performed by laparoscopy, trigger surprisingly high levels of postoperative pain [1]. To reduce the incidence of severe pain, patients who have undergone surgery, especially procedures deemed to be minor, should be monitored more precisely. Postoperative pain relief should conform to specific procedures and recommendations [2].

Narcosis (loss of consciousness), analgesia, and muscle relaxation are three major components of anesthesia. Unlike the other components, the assessment of analgesia (or “antinociception”), in current clinical practice, is based on clinical signs that are not very specific, such as movement, lacrimation, tachycardia, or hypertension. Optimized analgesia should individualize the nociception/antinociception balance and optimize the prevention of these reactions to surgical stimuli. In recent years, several monitors have been developed to measure the nociception/antinociception balance [3, 4].

Among them, the Analgesia/Nociception Index (ANI) has been developed to assess intraoperative analgesia using variations of the relative parasympathetic tone [5]. The ANI is obtained using the ANI monitor^®^ (MDoloris Medical Systems, Lille, France). This non-invasive device records the ECG tracing from electrodes placed on the patient’s chest and displays an instant (ANIi) and a 2-min moving mean (ANIm) of the ANI. The Calculation of the ANI was previously described in detail [3, 6, 7]. Briefly, the ANI is an index ranging from 0 to 100 derived from the high-frequency component of heart rate variability modulated by the effect of respiration on the RR series representing relative parasympathetic tone, which reflects the nociception/antinociception balance [7, 8]. High ANI values (above 50) indicate predominant parasympathetic tone, as observed in cases of adequate analgesia [8, 9]. In case of nociception, the sympathetic tone increases and the parasympathetic tone decreases, leading to reduced ANI values (below 50) and hemodynamic reactivity [8, 9].

Variations in ANI reflect the changes in nociceptive stimulation during general anesthesia combining various narcotic (propofol, sevoflurane, or desflurane) and analgesic (fentanyl, sufentanil, and remifentanil) agents [5, 10–12]. The target ANI range value during general anesthesia is 50 to 80 in order to optimize the intraoperative administration of opioids, in particular, remifentanil [9].

Given its extremely rapid pharmacokinetic and pharmacodynamic properties (length of time to onset < 1 min and duration of action < 10 min), remifentanil, which has already been used in our earlier ANI studies, appears to be an analgesic agent that is particularly suitable for continuous monitoring of the nociception/antinociception balance [5, 9, 13–15]. However, the use of high doses of remifentanil may lead to the risk of chronic pain, which can be partly prevented by the administration of ketamine, or cause hypotension or intraoperative bradycardia warranting optimization of the intraoperative administration of this drug [16, 17].

In a previous study, we have highlighted the good performance of ANI recorded at the arrival in PACU for the assessment of immediate postoperative pain [14]. The area under the receiver operating characteristics curve (ROC AUC) was 0.86, with 78% sensitivity and 80% specificity to detect patients with moderate to severe pain, assessed with the Numerical Rating Scale (NRS) > 3.

In this study, we used desflurane as an inhaled narcotic agent since it has rapid pharmacokinetics and pharmacodynamics properties because of its low liposolubility [18]. These properties swiftly balance effective concentrations of desflurane during anesthesia and ensure good hemodynamic control and rapid elimination at the end of anesthesia to facilitate early extubation and recovery [18]. The target intraoperative effective expiratory concentration of desflurane can be estimated from the minimum alveolar concentration (MAC) of halogen-based substances [18]. The MAC is defined by the expiratory alveolar concentration of a halogen that reduces movement by 50% in response to a nociceptive stimulus in anesthetized patients. This concentration decreases with age but the age-indexed MAC overcomes this. Overall, a MAC ranging from 0.8 to 1.2 can obtain the same pharmacodynamic objective as a BIS between 40 and 60 (adequate narcosis and reduction in the risk of intraoperative memorization) simply and at lower cost (the use of BIS electrodes is a non-negligible cost in itself) [19, 20]. Conversely, all of the ventilators currently used comprise a gas analyzer and can measure the fraction of halogen agents inspired and expired as well as the MAC without incurring any additional cost.

Following this study showing that ANI could assess NRS > 3 in PACU, we hypothesized that ANI in the end of surgical procedure may also be able to predict immediate postoperative pain. Therefore, we conducted a study showing that after general anesthesia combining desflurane and remifentanil, an ANI value < 50 immediately after extubation predicted the onset of postoperative pain (NRS > 3) in PACU with 86% sensitivity and specificity [15]. This study showed that ANI assessed immediately after extubation exhibits good performance (ROC AUC = 0.89) to predict immediate postoperative pain.

### Objective {7}

Following this study, we hypothesized that immediate postoperative pain in PACU could be reduced in patients with low ANI after extubation using a morphine bolus. Consequently, we designed this controlled, randomized study to establish whether the prophylactic administration of morphine at the end of the procedure in patients at risk of postoperative pain (ANI < 50 immediately before extubation) could reduce the incidence of the latter by 50%. Our secondary objective was to determine whether the intraoperative use of goal-directed analgesia with an ANI target of 50 to 80 could improve intraoperative hemodynamics and postoperative outcome.

### Trial design {8}

The GOALDAN study is an international, multicenter, simple-blind, parallel, prospective, randomized, controlled, two-armed superiority trial. Patients are randomly assigned in a 1:1 ratio in the control group or in the experimental group.

## Methods: participants, interventions, and outcomes

### Study setting {9}

The patients will be recruited from the University Hospital of Clermont-Ferrand, Clermont-Ferrand, France, the Maisonneuve-Rosemont Hospital, Montréal, Québec, and the Pierre Oudot Hospital Centre, Bourgoin-Jallieu, France. Patients will be considered for inclusion if they meet the criteria defined below.

### Eligibility criteria {10}

Participants meeting the following criteria will be included: Adult patients undergoing elective surgery involving general anesthesia with intubation, having an *American Society of Anesthesiologists* (ASA) score of I to III, who have given their consent according to the methods described in article L1122-1-1 of the French Public Health Code, and affiliated to a social security scheme or benefiting from such a scheme.

Participants meeting one or more of the following criteria will be excluded: age under 18 years, elderly patients aged over 75 years [21], general anesthesia without intubation (laryngeal mask), intraoperative local anesthesia with neuraxial block (peridural or spinal block), opioid-free anesthesia, arrhythmia or presence of a pacemaker, outpatient surgery, cardiac or cerebral surgery, obstetric surgery (Cesarean section), surgery performed with local neuraxial or peripheral anesthesia alone, surgery performed in prone position, emergency surgery, endoscopic procedure or interventional radiology, chronic pain treated by opioids, surgery scheduled to take less than 1 h, autonomous nervous system disorder (epilepsy, history of transient ischemic attack or stroke, paraplegia, hemiplegia, orthostatic hypotension, dysautonomia), patient suffering from cardiogenic or septic shock, continuous perfusion of vasoactive agents (ephedrine, phenylephrine, adrenaline or noradrenaline), scheduled postoperative transfer to ICU (patient intubated) after surgery, person under guardianship or curatorship, pregnancy, and breastfeeding.

### Who will take informed consent? {26a}

During the preoperative visit, on the patient’s arrival in the hospital department on the day before surgery, the investigating physician will screen the patient for eligibility to participate, invite the patient to take part in the study and hand out the patient information leaflet, outline the trial (objectives, benefits, and constraints for the patient), and check that the NRS has been understood in order to evaluate postoperative pain. This NRS is routinely presented to all patients undergoing a surgical procedure.

On the day of the procedure, the investigating physician will collect the signed consent form after ensuring that the patient has understood the information leaflet, and recheck the inclusion and non-inclusion criteria.

Randomization will be carried out using the electronic case report form (eCRF) module (allocation of the randomization group and number), and presurgical data will be entered in the eCRF.

### Additional consent provisions for collection and use of participant data and biological specimens {26b}

This trial does not involve collecting biological specimens for storage.

### Interventions

#### Explanation for the choice of comparators {6b}

Patients will be randomly allocated to either the intervention group (ANI) or the control group (standard care only). Standard care was chosen as an appropriate comparator given that the intervention is proposed as an adjunct to routine care. Indeed, there is to date no recommendation for routine intraoperative analgesia monitoring.

#### Intervention description {11a}

In the control group, patients will receive general anesthesia with propofol, ketamine, and remifentanil (use of neuromuscular blocking agent left to the discretion of the investigator) for induction and desflurane and remifentanil (with or without neuromuscular blocking agent) for maintenance of anesthesia. Anesthetic drug dosing and administration will be left to the discretion of the investigator. Intraoperative Analgesia/Nociception Index (ANI) will be monitored; however, data will not accessible to the investigator but will be stored in the monitor intern memory for further analysis.

**Fig. 1:**
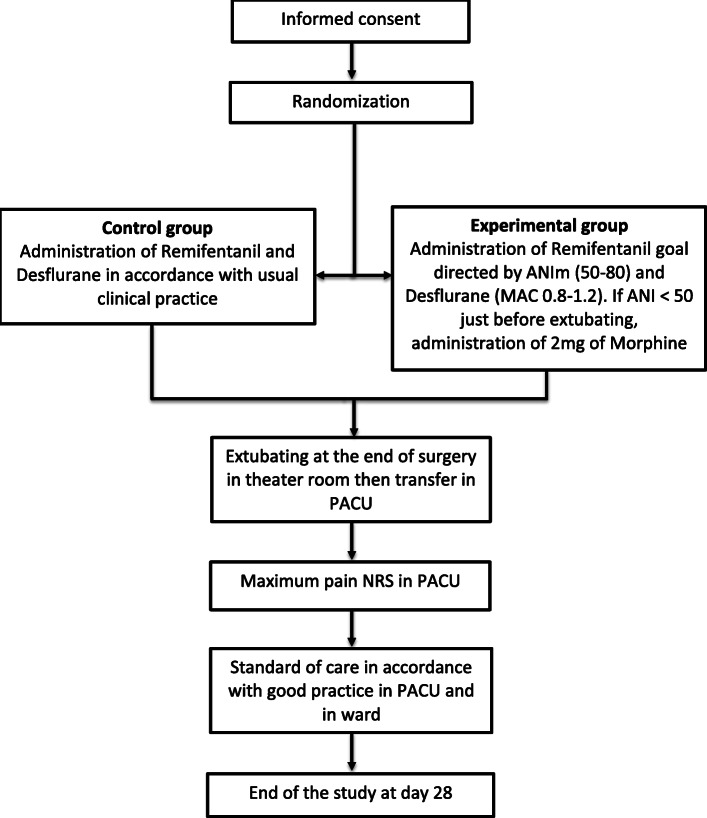
Study protocol diagram

In the experimental (ANI) group, patients will receive general anesthesia with propofol, ketamine, and remifentanil (with or without neuromuscular blocking agent) for induction and desflurane and remifentanil for maintenance with a specific algorithm (Fig. 1—study protocol diagram). The administration of remifentanil will be goal-directed by ANI, as well as the prophylactic injection of morphine at the end of surgery.

#### Criteria for discontinuing or modifying allocated interventions {11b}

All patients may leave the study at any time for any reason if they wish to do so without any consequences. Interventions will be discontinued if there are abundant adverse events (AEs) or serious adverse events (SAEs).

#### Strategies to improve adherence to interventions {11c}

Since patients in the ANI group will be on general anesthesia during the study, no specific strategy to improve adherence to intervention will be needed.

#### Relevant concomitant care permitted or prohibited during the trial {11d}

Authorized medicinal products are those normally used during general anesthesia. The patient’s routine treatments (antiplatelet drugs, β-blockers, and other antihypertensives, statins, etc.) will be managed in accordance with the current recommendations and the principles of good clinical practice. The following drugs are authorized within the scope of this trial: local anesthetics (levobupivacaine and ropivacaine), analgesics (ketoprofen, nefopam, paracetamol, tramadol), antiemetics (dexamethasone, droperidol, ondansetron), neuromuscular blocking agents (cisatracurium, succinylcholine), narcotics (desflurane, ketamine, propofol), and opioids (morphine, remifentanil).

The ANI calculation can be changed over 5 to 10 min following the administration of drugs affecting sinus cardiac activity (atropine, ephedrine, and phenylephrine mainly in the surgical context). If one or more of these drugs must be administered in the form of a bolus, this will be entered in the electronic case report form (eCRF), and the remifentanil dose will be maintained for the next 10 min without considering variations in ANI.

The continuous administration of vasopressors (ephedrine, phenylephrine, or noradrenaline) is prohibited during the study. If one or more of these drugs must be administered in the form of a bolus, this will exclude the patient from the study.

#### Provisions for post-trial care {30}

This study will not include any ancillary or post-trial care. There is no compensation budget item for this study.

### Outcomes {12}

The primary outcome is the immediate postoperative pain in PACU defined by an NRS > 3 during PACU stay. The secondary outcomes are as follows:

- Maximum pain score on the first day after surgery (0 to 10 rating scale). The NRS will be assessed every 6 h for the first 24 h (T0 = time of arrival in the PACU).
- Overall pain management satisfaction score on day 1 after surgery (0–10 NRS).
- Morphine dose level in PACU (mg).
- Postoperative nausea and vomiting in PACU.
- Total dose of morphine administered on the first day after surgery (mg).
- Duration of PACU stay (time taken to obtain an Aldrete score ≥ 9) (min) [22].
- Patient destination after surgery (surgical unit, follow-up care, ICU).
- Incidence of postoperative complications on the first and seventh day after surgery for hospitalized patients using the *Post-Operative Morbidity Survey* (POMS) (Annex 1) [23].
- Duration of hospital stay (days).
- Mortality at 28 days.

#### Participant timeline {13}

The participant timeline is shown in Table 1.

**Table 1:** Participant timeline

Time point**	Study period						
	Enrollment	Allocation	Post-allocation				Close-out
	D-1	D0	D0	D1	D7	D28	D28
**Enrollment**							
**Eligibility screen**	X						
**Informed consent**	X						
**Inclusion and non-inclusion criteria**	X						
**Allocation**		X					
**Interventions**							
**Control group**			X				X
**Experimental group**			X				X
**Assessments**							
**Clinical data**			X				
**Pain intensity (NRS scale)**			X	X		X	X
**Dose of analgesics**			X	X			
**Postoperative complications (POMS)**				X	X		
**Vital status**						X	X

#### Sample size {14}

Given the fact that immediate postoperative pain can affect 30% of patients [1], a total of 161 patients per group (322 patients) will give 90% power using a bilateral test with a *p* value of 5% to detect a 50% reduction in the incidence of immediate postoperative pain (NRS ≤ 3) in patients guided by ANI. Given the specific features of this protocol (essentially the direct transfer from operating theater to continuous monitoring or ICU, use of continuous vasopressors), the final decision is to include a total of 380 patients (190 per group). An interim analysis will be scheduled halfway through the enrolment period (95 patients per group). The *p* value used to decide withdrawal from the trial on efficacy grounds is set at 0.003 (correction of the O’Brien-Fleming alpha-spending function).

#### Recruitment {15}

Participating centers, Clermont-Ferrand University hospital (primary site), Pierre Oudot Hospital Center, and Maisonneuve-Rosemont Hospital, have been chosen for their sufficient number of surgical cases and the knowledge of investigators in ANI-guided remifentanil administration. To achieve a fast and easy enrollment, a dedicated password-protected website (Research Electronic Data Capture, RedCap^®^, Nashville, TN, USA) will be used to register consent, inclusion, and randomization.

### Assignment of interventions: allocation

#### Sequence generation {16a}

Randomization will be performed over the REDCap^®^ website (https://redcap.chu-clermontferrand.fr) to allow immediate allocation. The randomization list is balanced by blocks of variable and undisclosed size and stratified per center and per type of surgery. Patients will be randomized at day 0, after written informed consent is obtained and just before surgery. Each patient will be given a unique patient number and a unique randomization number. It is not possible to change group after randomization.

#### Concealment mechanism {16b}

The concealment mechanism is provided by REDCap^®^, which allows the user to preform randomization without the possibility of knowing the outcome beforehand. While blinding clinicians to treatment allocation is desirable, it is not deemed feasible for this study. We minimize bias through allocation concealment.

#### Implementation {16c}

The allocation sequence will be generated by a person not involved with enrollment or future analysis. Participants will be screened and randomized on admission to the operating room by a site investigator or member of research support staff.

### Assignment of interventions: blinding

#### Who will be blinded {17a}

Physicians and anesthetic nurses in the theater room cannot be blinded for the randomized arm. Physicians, nurses in PACU and in the surgical ward, and patients will be kept blinded for the randomized arm. Data analysts will be blinded for the randomized arm. Outcomes are mainly objective abstracted from electronic data to reduce bias.

#### Procedure for unblinding if needed {17b}

The design is an open label with only the statisticians/data analysts being blinded so unblinding will not occur.

### Data collection and management

#### Plans for assessment and collection of outcomes {18a}

All study data will be collected by an investigator of the research staff at each site using an eCRF and stored in a password-protected, traceable database (REDCap^®^) managed by the promotor (Clermont-Ferrand University). All parameters are defined in a data dictionary detailing the way in which data should be collected. The study promotor will ensure site visits for data monitoring, timely resolution of queries, and correction of errata during quality control checks.

#### Plans to promote participant retention and complete follow-up {18b}

The intervention is to be administered soon after operating theater admission and will last a short duration of time, likely while participants are unconscious. Patients will be approached once conscious and practical to do so, to inform them of study progress and follow-up required by a phone call at day 28. Obtaining data from medical records will ensure complete follow-up until patient discharge.

#### Data management {19}

Study data will be collected and managed using REDCap^®^ electronic data capture tool hosted and managed by the promotor (Clermont-Ferrand University) [24]. REDCap^®^ allows for a secure support data capture for research studies, providing audit trails for tracking data manipulation and export procedures, automated export procedures for common statistical software and procedures for data integration and interoperability with external sources.

#### Confidentiality {27}

According to the French and Canadian laws, each personnel who have a direct access to data will have to take all necessary precautions to ensure confidentiality of information related to experimental drugs, trials, and participants especially about their identity. Personnel as an investigator are bound by professional secrecy. The anonymity of each participant is ensured by a code number and initial of participants on the research documents. Identifying documents such as consent forms will be kept in locked rooms that may only be accessed by authorized personnel.

#### Plans for collection, laboratory evaluation, and storage of biological specimens for genetic or molecular analysis in this trial/future use {33}

Please see item 26b, this trial does not involve collecting biological specimens.

## Statistical methods

### Statistical methods for primary and secondary outcomes {20a}

The main analysis will take the form of an intent-to-treat procedure using the Stata^®^ (version 13, StataCorp, College Station) and R (http://cran.r-project.org/) software; all of the statistical tests will be carried out with an α value of 5%. The continuous variables will be presented as mean and standard deviation, provided that distribution is normal (Shapiro-Wilk test, if required). In the event of an anomaly, they will be presented as median, quartiles, and ranges. Qualitative variables will be expressed in numbers and related percentages. Where feasible, graphs will accompany these analyses. Intergroup comparisons will be routinely carried out (1) without adjustment (2) by adjusting factors, the distribution of which might not be balanced between the groups, despite randomization.

The patients will be described and compared between the groups at baseline, according to the following variables: compliance with eligibility criteria, epidemiological characteristics, clinical characteristics, and possible treatments. The initial comparability of both arms will be assessed on the basis of the principal characteristics of participants and potential factors linked to the primary endpoint. Any difference between the two groups in terms of one of these characteristics will be determined based on clinical as opposed to purely statistical considerations. Protocol deviations, patients distributed according to these deviations, and reasons for withdrawal will also be described. The number of patients enrolled and the enrolment graph will be presented per group.

Since a 50% reduction in the incidence of immediate postoperative pain in PACU patients receiving ANI-directed intraoperative analgesia ranging from 50 to 80 must be highlighted, the primary endpoint (percentage of patients with NRS > 3) will be compared using the *x*^2^ paired test or Fisher’s exact test, if applicable.

Secondly, this analysis can be completed in a multivariate situation using a generalized, linear mixed model approach to logistics, considering inter- and intra-site variability. The covariables will be considered in terms of clinical relevance (other treatments, continuous use of vasopressors, type of surgery) and the results of previous univariate analyses. The results will be expressed in terms of related odds ratios and 95% confidence intervals.

For the other qualitative parameters (percentage of patients with a NRS > 3 on the first day following surgery, postoperative complications on the first and seventh days after surgery, and patient destination following surgery (surgery unit, continuous care, intensive care), analyses relating to the secondary objectives will focus on the same approach as that previously recommended for the primary endpoint.

The quantitative secondary endpoints (maximum pain score on the first day following surgery, PACU dose levels of morphine, total dose of morphine on the first day following surgery, duration of PACU (time taken to obtain an Aldrete score ≥ 9), duration of hospital stay) will be compared between the groups using Student’s *t* test or Mann-Whitney’s non-parametric test if the *t* test conditions are not respected (normality study, homoscedasticity studied using the Fisher-Snedecor test). The results will be expressed in terms of effect size and 95% confidence interval. These analyses can be completed a second time in a multivariate situation using linear regression models. The covariables will be considered in terms of clinical relevance and the results of previous univariate analyses. The results will be expressed in terms of related regression coefficients and 95% confidence intervals.

Survival on day 28 post-surgery will be estimated according to the Kaplan-Meier curve and compared between the randomization groups using the log-rank test in a univariate situation and Cox’s model in multivariate analysis. The results will be expressed in terms of the immediate relative risk ratio and 95% confidence intervals.

Part of the analyses concerning the secondary endpoints will be exploratory in nature and should lack statistical power. As discussed by Feise in 2002, adjustment of the *p* value will not be proposed routinely but on a case-by-case basis depending on clinical as opposed to purely statistical considerations [25].

### Interim analyses {21b}

An interim analysis will be scheduled halfway through the enrollment period (95 patients per group).

### Methods for additional analyses (e.g., subgroup analyses) {20b}

Additional analyses are not planned.

### Methods in analysis to handle protocol non-adherence and any statistical methods to handle missing data {20c}

Data will be analyzed using an intention-to-treat methodology. A sensitivity analysis will be proposed to define the attrition level and statistical nature of the missing data in order to propose the most appropriate imputation method (maximum bias or multiple imputation), if applicable.

### Plans to give access to the full protocol, participant-level data, and statistical code {31c}

The full protocol, participant-level dataset, and statistical code will be available in accordance with Clermont-Ferrand University data sharing protocols.

### Oversight and monitoring

#### Composition of the coordinating center and trial steering committee {5d}

The trial steering committee is composed of investigators from the departments of anesthesiology and intensive care. The chair of the steering committee, through the Clermont-Ferrand University Hospital Centre, will be coordinating this study.

#### Composition of the data monitoring committee, its role, and reporting structure {21a}

The research assistant of Clermont-Ferrand University Hospital Centre will be coordinating the data monitoring committee. Serious adverse events will be reviewed regularly by the study steering committee and reported to the ethics committee.

#### Adverse event reporting and harms {22}

Adverse events will be collected in accordance with the Ethics Committee guidelines. Serious adverse reactions will be reported to the institutional research support services at each site. Significant safety issues will be reported to the Ethics Committee.

#### Frequency and plans for auditing trial conduct {23}

After each site is activated and has enrolled at least five patients, site monitoring of the consenting process, protocol adherence, and data collection will be conducted. At the end of this study, all sites will be monitored for protocol adherence and completion of data collection.

#### Plans for communicating important protocol amendments to relevant parties (e.g. trial participants, ethical committees) {25}

Any amendments to the protocol must be qualified as substantial or non-substantial. If they are deemed to be substantial, they must be reviewed by the ethics committee. Furthermore, any extension to the trial (in-depth amendment to the treatment design or cohort enrolled, prolonged administration of treatment, and/or therapeutic procedures not initially provided for in the protocol) must be classed as a new trial. Any deviations from the protocol will be fully documented using a breach report form.

#### Dissemination plans {31a}

The study shall be the subject of a declaration before being included on the internet site and made accessible to the general public [ClinicalTrials.gov]. The sponsor owns the data and this information cannot be used by or transmitted to a third party without the sponsor’s prior consent. The names of the sponsor, all of the investigators involved in patient recruitment or follow-up, methodologists, biostatisticians, and data managers participating in the trial will be mentioned when the main results are published. International regulations governing writing and publication shall be taken into account [26]. For the main publication, the scientific coordinator will be mentioned as the first author and the coordinating investigator as the last author.

## Discussion

The GOALDAN study has been designed to be pragmatic. Although anesthesia protocol is well codified in the experimental group, it remains however highly permissive concerning dose of anesthetics, analgesia, and nausea management. The risk of hyperalgesia induced by remifentanil is counterbalanced by the administration of ketamine [16].

The possibility to use morphine as usual analgesia management 30 to 60 min before the end of surgery could be a bias, but the difference in clinical practice in each center should reduce this bias. In the same way, the possibility to perform preoperative regional analgesia could decrease the incidence of patients with ANI < 50 at the end of surgery but it is ethically inconceivable not to offer this type of analgesia whenever possible.

The scope of the trial and its conditions comply with daily practices. The investigators responsible for applying the protocol on each of the sites participating in the trial have clinical trial experience. The measures taken to establish the endpoints will be carried out by appropriately trained professionals on each site (anesthetist physician or resident, state-registered nurse anesthetist, clinical research associate). The study period is set at 2 years to ensure recruitment consistency among sites participating in the trial. Two patients are anticipated to be enrolled per site and per month to ensure the feasibility of this protocol.

Because of the paucity of well-conducted trials, the authors believe that a randomized-controlled trial will improve the evidence for using analgesia monitoring during general anesthesia and strengthen the current recommendations for intraoperative analgesia management.

## Trial status

Inclusions started in June 2019. Enrollment is ongoing. The last version of the protocol is 4.0. Inclusions are expected to be completed in August 2022.

## Abbreviations

ANI: Analgesia/Nociception Index
ASA score: American Society of Anesthesiologists Score
eCRF: Electronic case report form
ICU: Intensive care unit
MAC: Minimal alveolar concentration
NRS: Numerical Rating Scale
PACU: Post-anesthesia care unit
POMS: Postoperative morbidity survey
